# Supplementary figures and images for: Exploring the phylogenetic diversity and antimicrobial activity of non-aureus staphylococci and mammaliicocci isolated from teat apices of organic dairy cows
Source: Front Microbiol. 2025 Sep 1;16:1614488. doi: 10.3389/fmicb.2025.1614488 (PMC12434115; doi:10.3389/fmicb.2025.1614488)

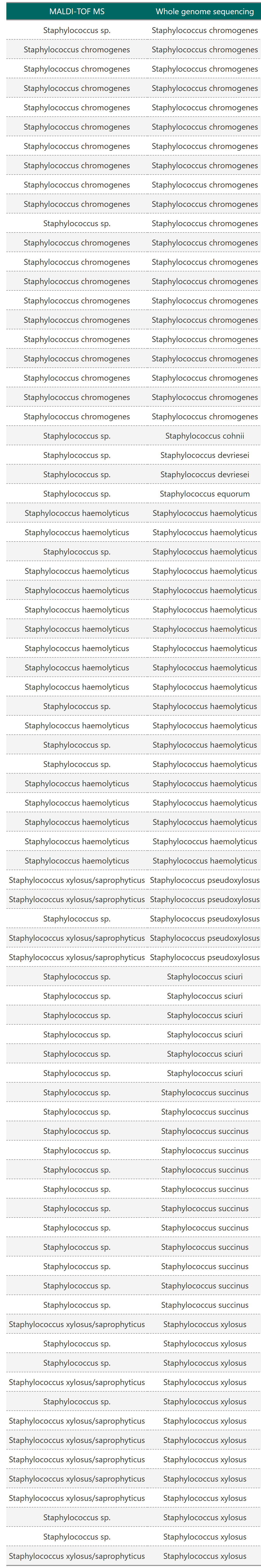

Supplement: Supplementary file 1 [file Image_1.png]
